# The anterior intercostal artery perforator flap in immediate oncoplastic breast reconstruction: current applications and future perspectives

**DOI:** 10.3389/fonc.2026.1765679

**Published:** 2026-02-06

**Authors:** Weijie Kong, Zhiyao Wang, Yang Liu, Jie Pei, Fan Guo

**Affiliations:** 1 Department of Breast Surgery, Third Hospital of Shanxi Medical University, Shanxi Bethune Hospital, Shanxi Academy of Medical Sciences Tongji Shanxi Hospital, Taiyuan, Shanxi, China; 2 Department of Plastic Surgery, Third Hospital of Shanxi Medical University, Shanxi Bethune Hospital, Shanxi Academy of Medical Sciences Tongji Shanxi Hospital, Taiyuan, Shanxi, China; 3 Department of General Surgery, Third Hospital of Shanxi Medical University, Shanxi Bethune Hospital, Shanxi Academy of Medical Sciences Tongji Shanxi Hospital, Taiyuan, Shanxi, China

**Keywords:** anterior intercostal artery perforator flap, breast reconstruction, breast-conserving surgery, immediate reconstruction, perforator flap

## Abstract

In recent years, the anterior intercostal artery perforator (AICAP) flap has gained increasing attention for its minimal donor-site morbidity, natural contour, and excellent scar concealment. This review begins by outlining the vascular anatomy of the anterior intercostal perforators, including their distribution, perfusion characteristics, and key considerations for preoperative imaging. We then summarize the indications and limitations of other commonly used chest wall perforator flaps, such as the lateral intercostal artery perforator (LICAP) and thoracodorsal artery perforator (TDAP) flaps to contextualize the unique role of AICAP in partial breast reconstruction. Building on this foundation, we focus on the surgical design, harvest dimensions, arc of rotation, and anatomical relationship between AICAP flaps and the inframammary fold. Compared with traditional local flaps, AICAP flaps offer reliable vascularity, excellent tissue compliance, minimal donor-site disruption, and low rates of postoperative complications. Their location within a natural skin crease also allows the resulting scar to remain well concealed. These features make AICAP particularly suitable for precise reconstruction of defects in the lower and lower-inner breast quadrants, where long-term aesthetic stability is often difficult to achieve. Nonetheless, standardized indications, anatomical variability, limited sample sizes, and a lack of long-term follow-up continue to constrain the strength of current evidence. Overall, this review synthesizes the anatomical basis, technical considerations, clinical advantages, and existing limitations of the AICAP flap. We further highlight emerging directions—including image-guided perforator mapping, personalized flap design, and long-term outcome assessment—to support the development of more standardized and reproducible clinical pathways for AICAP-based breast reconstruction.

## 1 Introduction

According to the 2022 report from the International Agency for Research on Cancer (IARC), breast cancer has become the most frequently diagnosed malignancy among women worldwide, accounting for approximately 23.8% of all new cancers and exceeding 2.31 million cases annually (1). In China, an estimated 357,200 new cases were recorded in the same year, ranking second among female malignancies and showing a continued rise, particularly among younger women (2).

Surgery remains the principal treatment modality for early-stage breast cancer, with mastectomy and breast-conserving therapy (BCT) constituting the two primary surgical approaches (3). Multiple randomized controlled trials have demonstrated that BCT followed by radiotherapy provides comparable local recurrence and overall survival outcomes to mastectomy (4). When oncologically appropriate, BCT preserves the native breast contour and significantly improves psychological well-being and overall quality of life, making it the preferred option for eligible patients (5). However, direct approximation of glandular or cutaneous tissue after tumor excision in standard BCT frequently results in contour deformity, volume deficiency, and displacement of the nipple–areolar complex (NAC)—issues particularly pronounced in defects located in the lower or inner quadrants of the breast (6). Oncoplastic breast-conserving surgery (OBCS) was introduced to address these challenges. With oncologic safety equivalent to conventional BCT, OBCS allows for wider excisions while restoring breast shape and symmetry, thereby improving aesthetic outcomes (7). Clough et al. classified OBCS into volume displacement and volume replacement techniques (8). When the resection volume is <20% of the total breast volume, glandular reshaping usually suffices; when 20–50% of the breast is removed, volume replacement using adjacent tissue flaps or implants becomes necessary. Traditional volume replacement frequently relies on myocutaneous flaps, which—although capable of providing adequate bulk—inevitably sacrifice muscle function and leave conspicuous donor-site scars. Denervation-induced atrophy and adjuvant radiotherapy further exacerbate long-term volume loss. In recent years, muscle-sparing chest wall perforator flaps have gained increasing attention as alternatives for small-volume replacement. These include LICAP, AICAP, and TDAP flaps (9). These flaps are based on reliable local perforators, preserve muscular function, provide well-concealed scars, and demonstrate stable shape after radiotherapy. Among them, the AICAP flap is supplied by anterior intercostal perforators, offering a short pedicle and direct rotational arc that allow precise reconstruction of lower and lower-inner quadrant defects while preserving the pectoralis major muscle (10). Compared with other chest wall perforator flaps such as LICAP or LTAP, AICAP offers better tissue pliability and more favorable anatomical alignment for small-breasted patients and lower-quadrant reconstruction. Despite its promising potential, evidence regarding standardized indications, perforator variability, complication rates, and long-term aesthetic outcomes for AICAP remains limited. Therefore, this review aims to provide a comprehensive synthesis of the vascular anatomy, surgical principles, clinical advantages, and limitations of the AICAP flap, and to explore current technical challenges and future directions related to imaging-guided navigation, personalized flap planning, and long-term oncologic and aesthetic outcomes.

## 2 Literature search strategy

This review was conducted as a narrative synthesis of the available literature addressing the use of anterior intercostal artery perforator (AICAP) flaps in immediate reconstruction following breast-conserving surgery for breast cancer. A comprehensive search of major English-language databases, including PubMed, MEDLINE, NCBI, and Springer, was performed for studies published up to October 2025. The search strategy incorporated the keywords *“anterior intercostal artery perforator flap,” “AICAP,” “chest wall perforator flap,”* and *“oncoplastic breast-conserving surgery,”* as well as relevant combinations thereof.

Eligible publications included clinical studies, anatomical investigations, and technical reports describing the application of AICAP or related chest wall perforator flaps in breast-conserving or immediate reconstructive settings. Reference lists of included articles were also manually screened to identify additional relevant studies. Given the narrative nature of this review, no strict restrictions were imposed on study design or outcome measures. Instead, emphasis was placed on surgical technique, indications, complication profiles, aesthetic outcomes, and available follow-up data.

## 3 Anatomical basis and perforator localization

Hamdi et al. (11) first provided a systematic description of the intercostal artery perforator (ICAP) flap and its anatomical classification in 2004. Intercostal perforators originate from the intercostal vascular arcade, which is formed by anastomoses between the internal thoracic artery and the aorta. Based on their course, these perforators are categorized into dorsal (DICAP), LICAP, and AICAP. The anterior intercostal arteries supplying the first through sixth interspaces arise from the internal thoracic artery, whereas those in the seventh through ninth interspaces derive from its musculophrenic branch. Anterior perforators predominantly cluster along the parasternal region and the inframammary fold (IMF). They typically emerge 1–2 cm lateral to the sternal border, traversing the intercostal muscles and pectoralis major before entering the subcutaneous plane, where they supply the overlying skin and superficial pectoral fascia. The fifth and sixth interspaces demonstrate the highest frequency of clinically relevant perforators and are most commonly harvested for AICAP flaps. Once within the subcutaneous tissue, these perforators form an extensive anastomotic network that interconnects with adjacent perforators, creating a robust microcirculatory system that supports flap elevation and diverse flap designs. Venous drainage is primarily through the internal thoracic vein, supplemented by subcutaneous communicating veins connecting to tributaries of the axillary vein, ensuring stable venous outflow. In an anatomical–clinical correlation study, Carrasco-López et al. (12) demonstrated that the IMF can be divided into medial, central, and lateral thirds, with at least one usable anterior intercostal perforator present in each region across 14 cadaveric specimens. This finding underscores the consistency and reproducibility of the vascular pattern. Quantitative measurements further revealed that AICAP perforators have an average diameter of 0.42 ± 0.05 mm and can be dissected to a usable pedicle length of approximately 3.1 ± 0.36 cm. These characteristics allow the flap to be designed along the IMF in multiple configurations, providing reliable perfusion and considerable flexibility in shaping.

**Figure 1:**
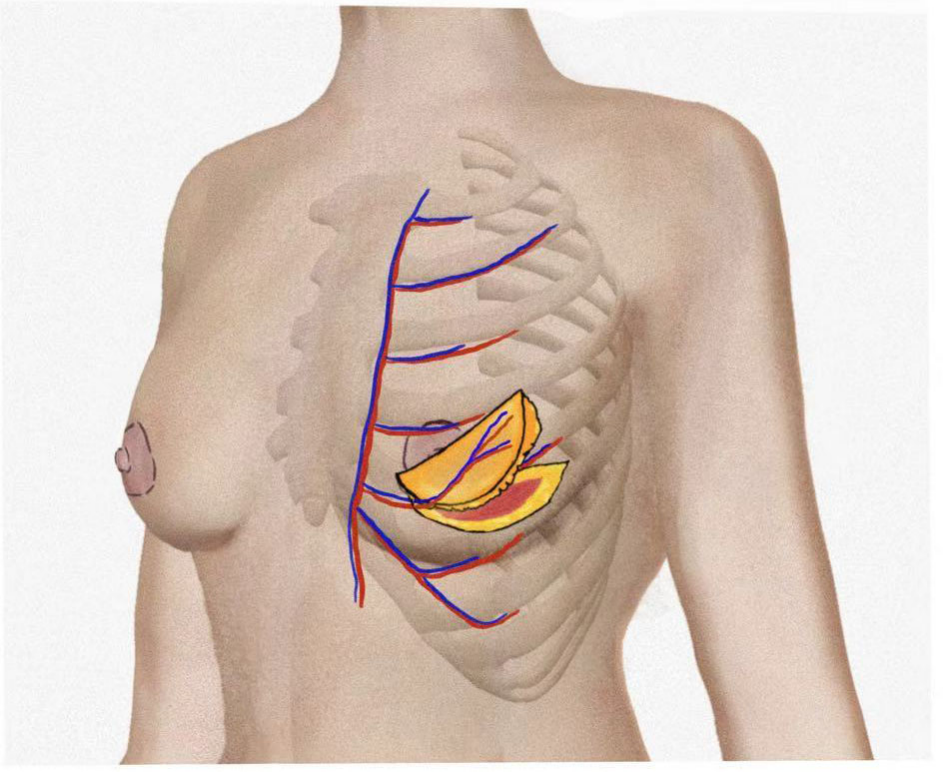
Anatomical overview of the anterior intercostal artery perforator (AICAP) flap.

Despite the relatively consistent course of AICAPs, their number, distribution, and caliber vary considerably among individuals. Carrasco-López et al. identified the lateral one-third of the IMF as a “perforator-rich zone,” where perforators are not only more numerous but also larger in diameter, suggesting an uneven distribution with anatomically favorable regions along the chest wall (12). Consequently, preoperative imaging is essential for optimizing flap design and minimizing intraoperative perforator injury. Current methods for AICAP localization rely heavily on imaging modalities, including handheld Doppler (HD), color Doppler ultrasonography (CDU), computed tomographic angiography (CTA), magnetic resonance angiography (MRA), and direct intraoperative visualization. HD is widely used due to its portability and real-time assessment capability; however, it is highly operator-dependent, fails to detect nearly half of perforators accurately, and may misidentify subcutaneous veins or small cutaneous branches as arterial perforators (13). CDU provides visualization of vessel diameter and flow characteristics and offers higher sensitivity and positive predictive value than HD, with less influence from body habitus, though its limited portability and steeper learning curve restrict routine use (14). CTA remains the current gold standard for perforator mapping, providing detailed visualization of perforator origin, course, and relationships with adjacent structures. Large-series CTA studies of chest wall perforators have reported detection rates of 87%–99%, making it particularly valuable for preoperative evaluation in complex cases (15). Its limitations include radiation exposure, contrast administration, and the inability to provide real-time intraoperative assessment. Therefore, small-incision direct visualization during surgery continues to serve as the definitive method for confirming perforator reliability, especially when imaging findings are equivocal. The anatomical characteristics of AICAP perforators are illustrated in **Figure 1**.

## 4 Indications, surgical technique, and postoperative management of the AICAP flap

### 4.1 Indications and contraindications

Clinical experience and existing reports suggest that the AICAP flap is particularly well suited for Asian women, especially those who are strongly motivated to preserve the breast, have small-to-moderate breast volume, mild ptosis, and clearly defined postoperative aesthetic expectations (16, 17). In terms of tumor location, AICAP is most frequently applied to lesions in the lower quadrant, periareolar region, and the area directly inferior to the NAC—zones that are prone to contour depression, nipple traction, and “bird-beak” deformities after standard breast-conserving excision due to inadequate soft-tissue support (18, 19). By providing direct volume replacement, AICAP effectively corrects these defects and restores breast contour and symmetry. With regard to tumor size and breast volume, AICAP is generally considered appropriate for T1–T2 breast cancers in patients with small-to-moderate breast size, where typical resections involve 20–30% of the breast. However, this threshold is not absolute. In small-breasted patients—particularly when lesions are located in the lower-inner quadrant, where the risk of postoperative deformity is inherently high—AICAP may be employed prophylactically even when the resection volume is <20% to better preserve breast shape (20, 21). Breast morphology also influences suitability. Patients with mild ptosis (grade I–II) derive particular benefit, as favorable soft-tissue elasticity and skin tension support long-term contour stability. Moreover, the IMF-based incision of the AICAP flap is easily concealed within natural folds or under clothing, and mild ptosis further enhances scar camouflage, reducing postoperative visibility concerns and anxiety. A few reports have also described the use of AICAP in conjunction with implants for total breast reconstruction, as well as its role in non-oncologic breast reshaping procedures (22, 23). As operative techniques continue to advance and multimodal reconstructive strategies evolve, the indications for AICAP are expected to expand further.

Contraindications to the AICAP flap are determined by tumor stage, patient comorbidities, and local anatomical factors. Absolute contraindications include T3–T4 tumors, multifocal or inflammatory breast cancer, active infection, and severe systemic disease that precludes safe anesthesia. Prior chest wall irradiation or thoracic surgery may disrupt perforator pathways; when preoperative imaging demonstrates a target perforator diameter <0.5 mm, an aberrant course, or poor flow signals, the risk should be carefully assessed and alternative reconstructive options considered (24–26). Local tissue conditions also warrant attention. Patients with thin skin, poor elasticity, or pre-existing scars may experience increased wound tension, predisposing them to dehiscence or compromised flap perfusion. Systemic factors such as diabetes and smoking further impair microvascular circulation and wound healing, increasing the risks of flap compromise and postoperative complications; these require meticulous perioperative management (27). Overall, successful use of the AICAP flap depends on thorough preoperative imaging-based assessment of perforator quality, optimization of the patient’s systemic condition, and careful selection of appropriate indications.

### 4.2 Preoperative evaluation: tumor localization and perforator selection

Preoperative assessment for AICAP reconstruction must account for both the extent of tumor excision and the vascular reliability of the selected perforator. Imaging evaluation—typically incorporating breast ultrasonography, mammography, and MRI—is essential for defining tumor location, margin characteristics, and its relationship to the glandular tissue, skin, and pectoral musculature. The planned resection generally includes the tumor itself along with a 1–2 cm margin of surrounding normal tissue, and the anticipated excision volume can be quantified using the ratio of “resected volume to total breast volume,” which informs subsequent flap design. In addition, the condition of the skin overlying the operative breast must be assessed, including elasticity, the presence of scars, and subcutaneous fat thickness, to determine both the suitability of the donor tissue and the likelihood of achieving stable postoperative contour.

**Figure 2:**
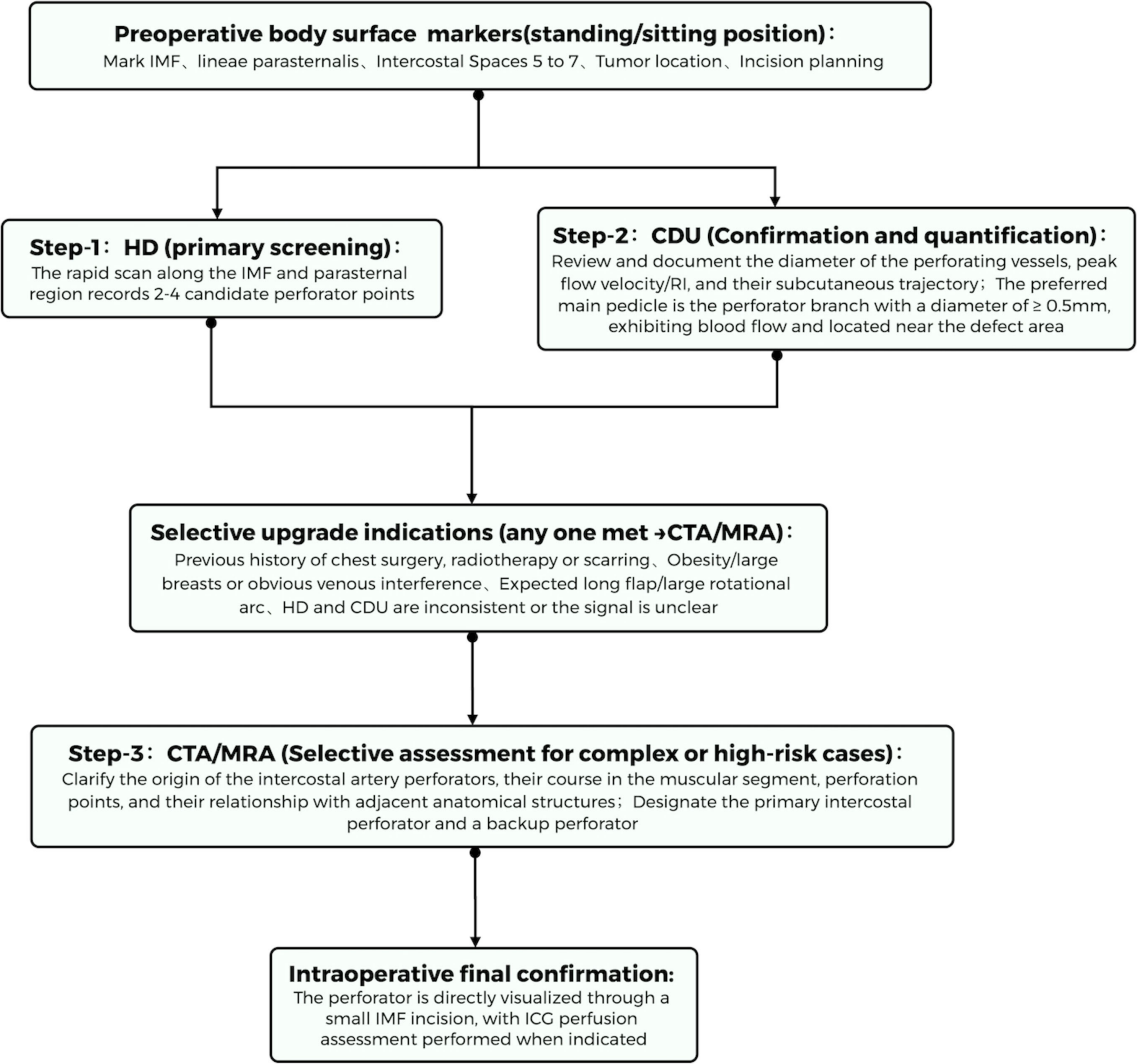
Graduated preoperative imaging workflow for AICAP perforator localization and selection. Surface landmarking is followed by handheld Doppler (HD) screening, color duplex ultrasonography (CDU) confirmation and quantification, selective CTA/MRA mapping when indicated, and final intraoperative visual confirmation.

Accurate perforator localization is a critical determinant of the safety and reproducibility of AICAP-based volume replacement. According to published data, approximately 76.2% of the blood supply to the lower breast quadrant originates from anterior intercostal perforators, and perforators with a diameter ≥0.5 mm are considered clinically significant (28). These perforators are most commonly found within 1–3 cm lateral to the sternal border in the fifth to seventh intercostal spaces, with an average diameter of 0.5–1.2 mm, providing reliable perfusion (15). Based on the marked anatomical variability of AICAP perforators and the complementary strengths and limitations of different imaging modalities, a stepwise preoperative imaging algorithm may be adopted in clinical practice to balance perforator detection accuracy with cost control and minimization of radiation exposure. This graduated approach consists of surface landmarking → initial screening with handheld Doppler (HD) → confirmation and quantification with color duplex ultrasonography (CDU) → selective high-resolution mapping with CTA (or MRA) → final intraoperative visual confirmation (**Figure 2**).

Preoperatively, surface markings of the inframammary fold (IMF), parasternal line, and target intercostal spaces are performed with the patient in the standing or seated position. HD is then used for rapid screening of the IMF and parasternal regions to identify and record two to four candidate perforators. CDU is subsequently employed to confirm arterial perforators and to quantify vessel diameter and hemodynamic parameters. Perforators with a diameter ≥ 0.5 mm, robust flow signals, and proximity to the anticipated defect are preferentially selected as the dominant pedicle, thereby reducing rotational tension and improving the predictability of volume replacement.

In patients with prior chest wall surgery or radiotherapy, obesity or large breast volume, anticipated long flap length or wide rotational arc, or when HD and CDU findings are inconclusive or discordant, selective escalation to CTA is recommended for three-dimensional precise mapping and identification of backup perforators, enhancing the reliability of preoperative planning and reducing the need for intraoperative modification (29). When CTA is contraindicated or when further reduction of radiation exposure is desired, MRA represents a feasible alternative for perforator visualization and surgical planning (30). In addition, assessment of the patency of the internal mammary artery trunk should be incorporated into preoperative evaluation to exclude significant stenosis or occlusion and to ensure safe flap perfusion and long-term volume stability.

Ultimately, intraoperative direct visualization of the perforator through a limited IMF incision (with adjunctive perfusion assessment when necessary) remains the final validation step in all cases. If intraoperative findings reveal suboptimal perforator quality, strategies such as reducing flap dimensions, converting to dual-perforator supply, or preserving a fascial bridge may be employed to augment perfusion. When these measures are insufficient, timely modification of the reconstructive plan—transitioning to alternative chest wall perforator flaps or combining with fat grafting and/or implant-based reconstruction—should be undertaken to ensure oncologic safety and reconstructive reliability (26).

### 4.3 Surgical technique and key technical considerations

#### 4.3.1 Surgical technique

Based on existing reports, the application of the AICAP flap in oncoplastic breast-conserving surgery can be broadly divided into five sequential stages: preoperative planning, tumor excision with cavity assessment, perforator dissection and flap harvest, flap shaping and transposition, and donor-site closure with drainage (15, 21, 31, 32).

Preoperative marking and incision design are performed with the patient in the standing or seated position to delineate the planned extent of tumor resection, the location of dominant perforators, and the inframammary fold (IMF). Flap size and incision configuration are determined according to the anticipated resection volume relative to total breast volume, skin laxity, and perforator position. The curvature of the flap should follow the natural contour of the IMF to allow primary donor-site closure and optimal scar concealment.

During tumor excision and cavity assessment, the patient is placed in the supine position with the ipsilateral arm abducted to 90°. Following sentinel lymph node biopsy and tumor resection, negative margins are confirmed on frozen section. Titanium clips are used to mark the tumor bed, and the residual cavity volume is assessed to guide final flap sizing. Perforator dissection and flap harvest are then performed through an IMF approach, with retrograde exposure of the anterior intercostal perforators. In most cases, the perforator with the largest caliber and a central entry point into the flap is selected as the dominant pedicle. The flap is elevated within the superficial fascial plane, preserving sufficient pedicle length to ensure a tension-free rotation.

The choice between a single- or dual-perforator design is guided by defect size, flap length and rotational arc, and intraoperative perfusion assessment. For small to moderate defects in which the flap can be transposed without tension, pedicle kinking, or compression, and with satisfactory distal bleeding, a single-perforator design is usually sufficient and facilitates simpler dissection. In contrast, for larger defects requiring a longer flap or wider rotational arc, or when distal perfusion appears borderline after transposition, preservation of a second perforator or a fascial bridge is recommended to provide additional perfusion redundancy (33). Other perforators deemed nonessential may be divided once tension-free positioning, absence of pedicle torsion, and adequate distal bleeding are confirmed.

**Figure 3:**
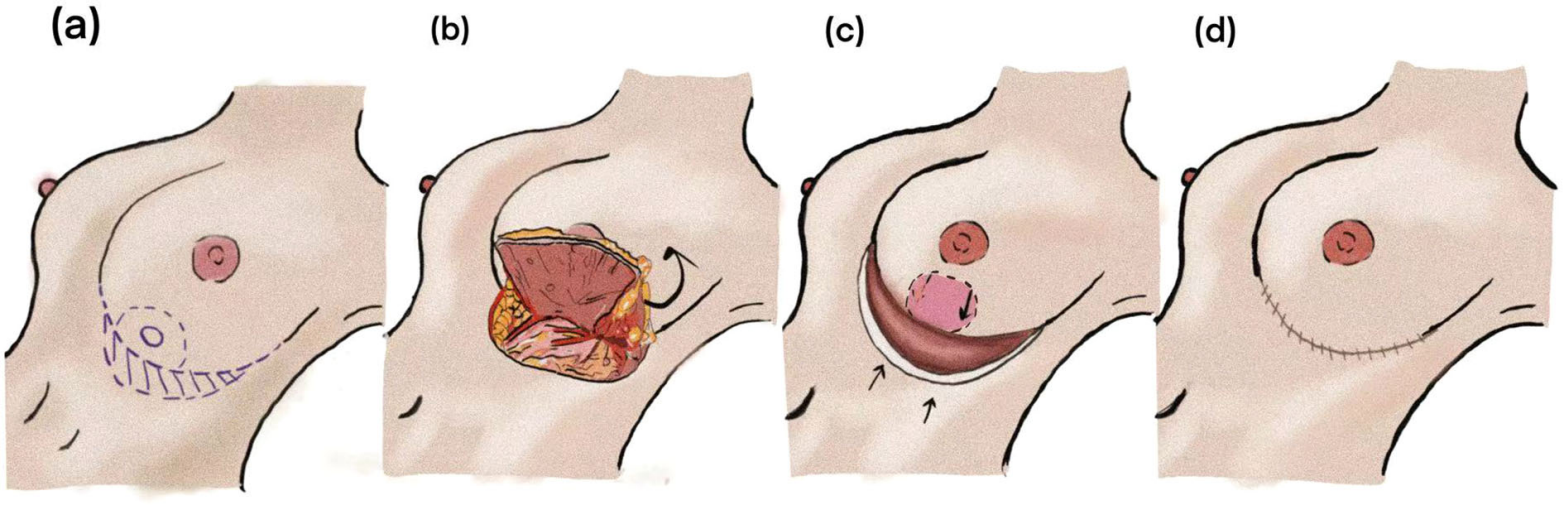
Surgical workflow of the AICAP flap in immediate oncoplastic breast reconstruction. **(a)** Mark the tumor location and anticipated resection area and design a crescent-shaped incision that follows the natural curvature of the inframammary fold. **(b)** Through the inframammary fold approach, perform tumor excision, then dissect the AICAP flap in a retrograde manner. After de-epithelialization, rotate the flap to fill the defect. **(c, d)** Adjust breast contour and symmetry, followed by layered closure of the incision.

After de-epithelialization, the flap is contoured and its ends are approximated according to the location of the dominant pedicle. The flap is then rotated into the defect without tension and secured to the tumor bed with interrupted sutures to achieve precise volume replacement of the inferomedial quadrant. Meticulous hemostasis is ensured, followed by primary donor-site closure and placement of a suction drain to monitor postoperative bleeding and seroma formation. The key steps of the AICAP flap procedure are summarized in **Figure 3**.

#### 4.3.2 Key technical considerations

##### 4.3.2.1 Intraoperative assessment of flap perfusion

Assessment of flap perfusion is an essential intraoperative step. This can be performed by direct visual inspection and gentle palpation or compression of the vascular pedicle; a palpable pulsation indicates adequate inflow. In addition, the presence of fresh bleeding from the flap surface after de-epithelialization is a reliable indicator of satisfactory perfusion. When tumor excision involves the overlying skin and results in a cutaneous defect, a small skin paddle may be preserved to resurface the defect, improve breast appearance, and allow postoperative dynamic monitoring of flap viability (34, 35). When available, indocyanine green (ICG) fluorescence angiography may be routinely used to provide an objective assessment of flap perfusion (36–38).

##### 4.3.2.2 Perforator preservation and pedicle tension control

During perforator dissection, a limited cuff of surrounding muscle fibers and interstitial tissue should be preserved to protect the perforator pedicle and reduce the risk of torsion or vasospasm (39). After flap transposition, the course and tension of the pedicle should be reassessed dynamically. A visibly straightened or stretched pedicle suggests excessive tension, which should be corrected by shortening the advancement distance or reducing the rotational arc to maintain unobstructed perfusion and prevent vascular compromise (40).

##### 4.3.2.3 Intraoperative breast shaping

Breast shaping should prioritize natural symmetry of the nipple–areolar complex (NAC) and the lower pole, with attention to long-term stability. The lower pole contour should demonstrate a gradual decrease in thickness from the NAC toward the inframammary fold (IMF); therefore, relatively greater tissue volume should be retained near the nipple–areolar region to achieve a natural projection and fullness. Intraoperative adjustments should be made with reference to the contralateral breast. Excessive fullness on the reconstructed side can be corrected by judicious flap trimming, whereas NAC displacement may be addressed by fine-tuning flap tension to restore symmetry in both breast contour and nipple position. Anticipating postoperative radiotherapy–related tissue contraction and fibrosis, deliberate preservation of additional lower-pole volume during shaping may help maintain long-term aesthetic quality (41).

##### 4.3.2.4 Maintenance of the inframammary fold

When AICAP flaps are used to repair defects after breast-conserving surgery, preservation of the IMF is critical to prevent inferior migration. Preoperatively, the IMF should be precisely marked in the standing or seated position and compared with the contralateral side, with the incision designed within or immediately along the upper margin of the IMF crease. Intraoperatively, IMF definition should not rely solely on skin closure. Instead, segmented deep anchoring sutures should be placed along the IMF, fixing the deep dermis or superficial fascia and the flap fascia to stable structures such as the deep chest wall fascia or costal perichondrium. Closure tension should be preferentially distributed to the deep layers to maintain IMF position and contour (42). For lower pole or inferolateral defects, additional anti-sliding fixation at the inferior edge of the flap and layered, planar filling may further reduce postoperative gravitational descent. Early postoperative use of a supportive bra can also help stabilize the IMF configuration during the healing phase (43).

### 4.4 Postoperative complications and management

Current clinical reports indicate that the overall complication rate of AICAP reconstruction is low, with most events involving hematoma, seroma, fat necrosis or liquefaction, delayed wound healing, infection, and small areas of partial flap necrosis (26, 44–46). The vast majority of these complications can be effectively managed through early recognition and standardized treatment algorithms, without compromising the reconstructive outcome or delaying subsequent oncologic therapy (26, 45). Hematomas and seromas are typically managed with close observation or needle aspiration in the early postoperative period, supplemented by appropriate compression and drainage. Meticulous perioperative wound care, thorough intraoperative irrigation, proper drain placement, and evidence-based antimicrobial strategies significantly reduce the risks of wound dehiscence and infection (13, 44, 45). Partial flap necrosis most commonly occurs at the distal or marginal portions of the flap and is typically associated with venous congestion or excessive tension. Most cases resolve with dressing changes, negative-pressure wound therapy, or delayed minor revision, achieving satisfactory healing (44, 46). Although extensive flap necrosis is rare, it requires prompt debridement; when local conditions permit, secondary reconstruction or conversion to an alternative technique may be considered (26, 45). Overall, postoperative complications following AICAP reconstruction are generally manageable events. With standardized perioperative management and timely intervention, both oncologic safety and aesthetic outcomes can be reliably maintained. To provide an overview of the current clinical evidence, representative studies evaluating the AICAP flap are summarized in **Table 1**.

**Table 1:** Summary of representative clinical studies involving the AICAP flap.

Study	Year	Study design	Sample size (N)	Indication/Quadrant	Technique characteristics	Main outcomes	Complications	Follow-up
Carrasco-López et al.	2017	Anatomical and prospective clinical series	14	Defects in lower-inner and adjacent quadrants	IMF-based AICAP; single perforator; Doppler mapping	High satisfaction; good symmetry; stable IMF	Hematoma (1); partial necrosis (1)	Mean 14 months (range 11–20)
Adler et al.	2023	Retrospective clinical series	16	Defects in upper/lower lateral quadrants; small breasts	IMF-based AICAP; medial perforator preferred	Satisfactory contour; no flap necrosis	Seroma (1); mild infection (1)	Median 3 months post-radiation (range 1–6)
Elmouty et al.	2023	Prospective clinical series	40	Lower-pole defects; small–medium breasts	IMF incision; Doppler mapping; adipofascial flap	82.5% excellent results; no flap loss	Fat necrosis (2); mild infection (2)	Mean 11.4 months (range 6–20)
Kollias et al.	2022	Single-centre observational cohort	30	Inferior-pole defects (4–8 o’clock)	IMF-based AICAP; ≥2 perforators; adipofascial flap	96.7% clear margins; no recurrence	Delayed healing (1); seroma (10); fat necrosis (1)	Median 65 months (range 12–89)
Agrawal et al.	2024	Retrospective single-centre cohort	47/150 (31.3%)	Defects mainly lateral; AICAP for lower-quadrant defects	Single-incision; Doppler mapping	High satisfaction; DFS 86.4%; OS 94.7%	Dehiscence (12.7%) seroma (7%); flap loss (1.3%);	Median 16 months (IQR 9–37)
Soumian et al.	2020	Prospective multicentre audit	25/112	Defects in outer and lower-inner/ central quadrants	Perforators at 6 o’clock; single-incision; Doppler mapping	Margin re-excision 13.4%; satisfactory aesthetics	Hematoma (4); dehiscence (1); seroma (1); fat necrosis (1)	Median 15 months
Galassi et al.	2025	Retrospective single-centre study	4/25 (16%)	Defects in upper outer, lower outer, lower inner quadrants	CTA/Doppler mapping; IMF-based AICAP	100% negative margins; high BREAST-Q	No complications	Follow-up at 1 week, 1 month, and 3 months; BREAST-Q at immediate postoperative and 3 months
Marruzzo et al.	2024	Retrospective study	4	Implant-related defects requiring coverage	IMF incision; Doppler mapping; single-perforator flap	Complete flap survival; no implant exposure	No complications; mild IMF asymmetry (1)	Mean 6 months (range 2–12)

AICAP, anterior intercostal artery perforator; IMF, inframammary fold; CTA, computed tomography angiography; BCS, breast-conserving surgery; DFS, disease-free survival; OS, overall survival; BREAST-Q, Breast Reconstruction Evaluation Assessment Scale.

## 5 Comparative review of other perforator flaps and limitations

Although AICAP offers a clear anatomical advantage for reconstruction of lower-inner quadrant defects, other chest wall perforator flaps—including LICAP, LTAP, TDAP, SEAP, and TDIP—are widely used for volume replacement in different breast quadrants. Each of these flaps has well-established applications in specific regions; however, their vascular supply, arc of rotation, and donor-site incision placement lead to notable differences in scar concealment, contour stability after radiotherapy, and overall surgical invasiveness. To define the distinct value of AICAP, it is essential to compare these commonly used flaps across key parameters such as indication range, donor-site morbidity, scar visibility, and long-term aesthetic outcomes. Such comparison helps delineate the precise role and advantage profile of AICAP within the broader chest wall perforator flap repertoire.

The TDAP flap, supplied by terminal branches of the thoracodorsal artery, is one of the earliest and most extensively validated local perforator flaps for reconstruction of defects in the lateral chest wall and upper-outer quadrant. It provides a long pedicle and ample tissue volume, making it suitable for moderate to large defects, and demonstrates relatively stable contour even in the setting of postoperative radiotherapy (47). Existing cohort studies indicate that TDAP achieves comparable negative-margin rates and local tumor control to other chest wall perforator flaps, with its oncologic safety well established (48). However, considerable anatomical variability in its perforators poses challenges. Preoperative localization is less predictable, and intraoperative dissection is more technically demanding than with more anteriorly based perforator flaps. Because the perforator travels through the latissimus dorsi muscle fibers, the dissection pathway is longer, and the flap may be restricted when axillary procedures are performed concurrently. Furthermore, the axillary-line incision tends to be more visible in slender patients. For lower-inner quadrant defects, TDAP must traverse a longer rotational arc to reach the target area, often requiring increased tension or additional contouring maneuvers, which limits its suitability for this region. These limitations have motivated surgeons to explore alternative perforator flaps harvested closer to the lower breast pole, with improved scar concealment and more favorable access for lower-quadrant reconstruction.

The lateral thoracic artery perforator (LTAP) flap is based on perforators arising anterior to the mid-axillary line from the second to fifth intercostal spaces. Its donor-site anatomy is relatively constant, providing reliable vascular supply for reconstruction of defects in the lateral and upper-outer breast quadrants, and it is one of the earliest techniques incorporated into the chest wall perforator flap repertoire. McCulley and colleagues first introduced the LTAP flap as a volume-replacement method following partial mastectomy. Recent studies have shown that a modified LTAP harvested through a vertical incision along the anterior axillary line achieves excellent flap viability, low donor-site morbidity, and satisfactory aesthetic outcomes in patients with small breast volume, with no adverse short-term oncologic signals (49, 50). However, the vascular territory of the LTAP flap remains primarily confined to the lateral chest wall. When defects involve the lower or lower-outer quadrants, the flap’s arc of rotation becomes limited and its available tissue volume may be insufficient to recreate the natural lower pole contour. In addition, incisions placed in the anterior axillary region provide less effective scar concealment than those hidden within the inframammary fold, and may interfere with axillary surgical access. These anatomical characteristics restrict the applicability of the LTAP flap in the reconstruction of lower-inner quadrant defects.

The LICAP flap is based on lateral intercostal perforators from the sixth to eighth interspaces. Its anatomy is relatively constant and the surgical technique straightforward, making it one of the most commonly used chest wall perforator flaps for reconstruction of lateral and lower-outer quadrant defects. In a comparative study of 83 patients, Hashem et al. (48) reported that LICAP and TDAP achieved similar negative-margin rates and local recurrence rates, while LICAP offered shorter operative times and better preservation of donor-site function. As a result, LICAP is often considered the preferred option for small to moderate lateral breast defects. However, conventional LICAP flaps are inherently constrained by their donor-site location and the limited arc of rotation. The resulting scar lies on the lateral chest wall, which is more visible in slender or younger patients; and when the flap is rotated medially or inferiorly, increasing tension and reduced tissue compliance may lead to flattening or traction deformity of the reconstructed lower pole. Modified techniques that access the flap through the retromammary plane can expand its reach to some extent (51), but the underlying vascular axis remains lateral, limiting its ability to provide the natural inferior pole support required for lower-inner quadrant reconstruction. In contrast, AICAP flaps—harvested from tissue directly adjacent to the lower-inner breast—offer a shorter rotational pathway, improved contour matching, and superior scar concealment, effectively addressing the “trouble zone” limitations that LICAP cannot fully overcome.

The superior epigastric artery perforator (SEAP) flap is supplied by perforators of the superior epigastric artery, which typically emerge along the upper mid-abdomen or near the level of the IMF. This allows a soft and compliant tissue paddle to be transferred superiorly without sacrificing the rectus abdominis muscle (52). Because the incision can be concealed along the IMF, SEAP offers better aesthetic outcomes than flaps harvested from the lateral chest wall and provides favorable tissue pliability for reconstruction of lower-inner quadrant defects. Several cohort studies have reported relatively stable postoperative contour following radiotherapy, with acceptable oncologic safety (53). However, the available tissue volume and perforator quality of the SEAP flap are highly dependent on individual anatomical variability. Abdominal scars, body habitus, and fat thickness may all restrict its applicability. For moderate to large defects, SEAP often fails to provide adequate support, and surgeons may need to rely on more complex composite designs—such as TDIP—to extend its reach and volume, thereby increasing technical demand and uncertainty. In comparison, AICAP flaps are harvested adjacent to the lower breast, providing sufficient volume and structural support while avoiding an additional donor site. This proximity grants AICAP a clearer advantage in adaptability and long-term stability for lower-inner quadrant reconstruction.

The thoracodorsal Intercostal Perforator flap (TDIP) flap is considered a composite extension of the TDAP concept, combining the thoracodorsal perforator with ipsilateral intercostal perforators to augment vascularity and increase the arc of rotation. This design allows coverage of challenging quadrants that cannot be reliably reached with a single lateral perforator. The theoretical advantage of TDIP lies in its dual perforator supply, which enhances distal perfusion and expands the flap’s rotational reach medially and inferiorly, making reconstruction of larger defects feasible (54). However, current evidence is limited, consisting mainly of small technical case series with substantially fewer documented cases than LICAP, LTAP, or TDAP, leaving the overall level of evidence relatively weak. The principal limitations of TDIP stem from its vascular and anatomical characteristics. First, the technique requires exposure of two separate perforator systems, significantly increasing operative time and technical complexity, making it impractical as a routine oncoplastic approach. Second, the donor site lies along the posterior axillary line near the axillary dissection field, where intraoperative traction may affect lymphatic drainage or jeopardize perforator flow. Third, because the flap must traverse a long and circuitous pathway from the lateral chest wall, tension, limited arc of rotation, and reduced pliability remain problematic when addressing anterior or lower-inner quadrant defects. As a result, successful contour restoration depends heavily on surgeon experience (44). In contrast, AICAP relies on a short pedicle, a focused operative field, and tissue harvested adjacent to the reconstructive zone, offering superior compliance and fewer anatomic constraints. These differences suggest that TDIP is better suited as a salvage or situational option for select anatomic challenges rather than a standard technique for lower-inner quadrant reconstruction.

**Figure 4:**
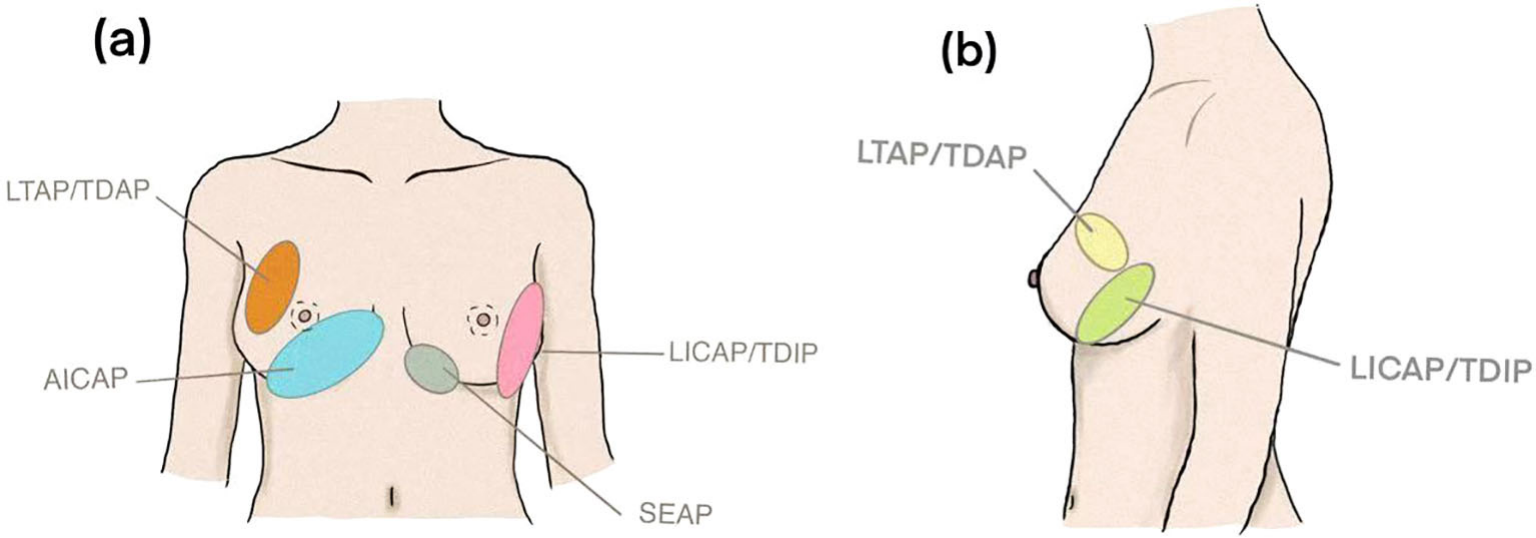
Typical flap selection for breast tumors located in different quadrants (schematic diagram). **(a)** Front view displaying labeled regions: LTAP/TDAP, AICAP, LICAP/TDIP, and SEAP. **(b)** Side view providing detailed visualization of the same areas.

Despite the increasing refinement of chest wall perforator techniques for immediate breast-conserving reconstruction, most established approaches still depend on lateral or posterior vascular supply. Their long rotational arcs and concentrated tension make them poorly suited for defects in the lower-inner quadrant and central breast. This anatomical constraint explains why LICAP, LTAP, and TDAP flaps perform predictably in the lateral quadrants yet frequently fail to provide adequate volume, tension control, or scar concealment for lower-pole restoration. These limitations have driven a shift from lateral-based designs toward the anterior–medial vascular territory of the breast. The introduction of the AICAP flap extends the perforator system beyond its traditional lateral dominance, addressing a long-standing anatomical gap in these “trouble zones.” Located immediately adjacent to the lower breast pole, AICAP offers a short rotational distance, superior tissue compliance, and reliable restoration of the natural inferior contour without compromising oncologic resection. The comparison of commonly used chest wall perforator flaps for partial breast reconstruction are summarized in **Table 2**. A schematic quadrant-based comparison of the typical indications for different perforator flaps is presented in **Figure 4**.

**Table 2:** Comparison of commonly used chest wall perforator flaps for partial breast reconstruction.

Flap type	Donor site/ Vascular origin	Indication quadrants	Rotation arc	Scar visibility	Post-radiation stability	Tissue pliability	Suitable breast size	Oncologic safety	Fat necrosis/ Reinterventions	Key advantages	Key limitations
LICAP	Lateral chest wall; 6th–8th Intercostal lateral perforators	Lateral/ lower-lateral	Moderate	Easily visible	Moderate	Moderate	Medium–large	Negative margins; no local recurrence^17,20^	Occasional fat necrosis; possible secondary revision	Reliable anatomy; simple dissection; muscle-sparing	Limited arc; lateral scar prominent
LTAP	Lateral thoracic Artery perforator; anterior axillary line (2nd–5th ICS)	Upper-lateral/ lateral	Moderate	Generally concealed	Good	Moderate	Small–medium	Negative margins; no recurrence^18-19^	Low fat necrosis; occasional secondary revision	Concealed donor site; good aesthetics	Suboptimal reach to lower-inner quadrant
TDAP	Thoracodorsal artery terminal perforator; through latissimus dorsi	Lateral/ upper-lateral	Moderate–wide	Moderate	Good	Slightly firm	Medium–large	Proven oncologic safety^16-17^	Low fat necrosis; possible secondary revision for bulk	Long pedicle; large volume; stable after RT	High perforator variability; complex dissection; axillary involvement
SEAP	Superior epigastric artery perforators (upper abdomen)	Lower/ lower-inner	Wide	Concealed in IMF/abdominal crease	Good	Soft	Small–medium	Good local control^21-22^	Fat necrosis risk when extended; occasional reintervention	Soft tissue; favorable IMF matching	Limited volume; high variability; imaging required
TDIP	Combined Thoracodorsal and intercostal perforators	Lateral extension to lower pole	Wide	Moderate	Good	Moderate	Medium–large	No recurrence reported^23-24^	Possible secondary revision in complex reconstructions	Dual blood supply; broad coverage	Technically demanding; axillary donor site; limited evidence
AICAP	Anterior intercostal perforators near IMF	Lower-inner/ subareolar	Short	Excellent (IMF concealed)	Excellent	Soft	Small–medium	High negative-margin rate; low complication rate^50-51^	Fat necrosis risk under high tension; occasional secondary revision	Natural contour; reliable perfusion; ideal for IMF defects	Limited volume; perforator variability; imaging-dependent

IMF, inframammary fold; ICS, intercostal space. Fat necrosis and reinterventions are summarized qualitatively based on published series. Post-radiation stability reflects reported contour preservation after radiotherapy.

## 6 AICAP reconstruction: clinical outcome evaluation

### 6.1 Oncologic and surgical safety

Current clinical evidence indicates that AICAP reconstruction provides not only reliable oncologic safety but also distinct procedural advantages in immediate breast-conserving reconstruction. Because the donor site lies adjacent to the lower-inner quadrant of the breast, AICAP allows wide tumor excision without compromising breast contour. This is particularly advantageous for small-to-moderate–volume breasts, in which achieving negative margins while preserving inferior pole support is essential for balancing oncologic clearance and aesthetic outcome. Both anatomical and clinical data support the stability of this approach. Carrasco-López et al. (12) demonstrated through combined anatomical mapping and clinical application that AICAP perforators follow a relatively consistent course and can be accessed through a focused operative field. All 14 patients in their immediate reconstruction series achieved complete flap survival without major complications requiring reoperation. Delays in adjuvant radiotherapy were not observed, ensuring timely continuation of systemic treatment. Similarly, Adler et al. (55) reported outcomes in 16 AICAP reconstructions, noting a 100% negative-margin rate and only minor complications manageable through conservative treatment, further confirming its short-term safety and reproducibility. Broader evidence from chest wall perforator flap (CWPF) cohorts provides midterm oncologic validation for this reconstructive family. In a study of 150 patients undergoing CWPF-based immediate reconstruction, Agrawal et al. (56) reported a predicted 5-year disease-free survival of 86.4% and overall survival of 94.7%, with very low positive-margin and re-excision rates. Most complications were mild and did not interfere with adjuvant therapy. Another study focusing on Asian women with small-to-moderate breast volume (16) echoed these findings, showing that CWPF combined with breast-conserving surgery maintains local tumor control while improving symmetry and aesthetic satisfaction. Comparative data indicate that AICAP achieves oncologic outcomes comparable to LICAP, LTAP, and TDAP, with similar rates of negative margins, re-excision, and short-term recurrence (16, 56), demonstrating no oncologic disadvantage. However, compared with TDAP—which requires a longer incision along the posterior axillary line and involves more variable perforators—AICAP offers a shorter dissection pathway, a concentrated operative field, and minimal disruption of axillary lymphatic structures. These features support smoother integration into standardized breast-conservation workflows and shorten the learning curve. Moreover, the smaller operative field reduces postoperative drain output, flap tension, and risk of fat necrosis, facilitating early recovery and timely radiotherapy. Taken together, the available evidence suggests that AICAP provides highly reproducible oncologic safety, low complication rates, and consistently favorable aesthetic outcomes. For patients seeking a balance between “adequate oncologic resection and aesthetic restoration,” AICAP offers a structurally advantageous reconstructive option—particularly suited for defects of the lower-inner quadrant and the region beneath the NAC.

### 6.2 Clinical performance and functional outcomes

As a true perforator-based technique, AICAP reconstruction minimizes donor-site morbidity and postoperative functional impairment. Because the flap harvest does not require division of major muscle groups such as the pectoralis major, latissimus dorsi, or serratus anterior, and because the donor site lies adjacent to the IMF with primary closure achievable in most cases, many of the donor-site complications associated with traditional myocutaneous flaps are effectively avoided. Compared with autologous options such as the transverse rectus abdominis myocutaneous (TRAM) flap or the latissimus dorsi (LD) flap, AICAP markedly reduces muscle-disruption–related risks. Previous cohort studies have shown that TRAM and LD flaps are often associated with abdominal wall bulging, reduced rectus abdominis strength, limited shoulder mobility, and donor-site seroma, whereas chest wall perforator flap (CWPF) techniques report low rates of functional impairment and rapid postoperative recovery (56, 57). Among CWPF approaches, AICAP also offers advantages over lateral perforator flaps such as TDAP and TDIP, which require incisions along the posterior axillary line. AICAP preserves muscular integrity and functional capacity while avoiding a large lateral incision and reducing potential interference with the axillary dissection field (58). Its more anterior–inferior harvest site shortens the flap’s transfer distance and facilitates seamless integration into standardized oncoplastic workflows. Taken together, AICAP demonstrates clear strengths in minimizing surgical trauma and preserving donor-site function. It circumvents the functional costs of traditional myocutaneous flaps while also overcoming the aesthetic disadvantages of some lateral CWPF techniques, making it an optimal choice for patients with small-to-moderate breast volume and those prioritizing aesthetic balance.

### 6.3 Aesthetic outcomes and patient satisfaction

Because the flap is harvested from the anterior intercostal perforator region adjacent to the breast, the subcutaneous fat thickness, skin color, and tissue consistency closely mirror those of the native breast. This allows the natural contour of the lower pole to be reconstructed without extensive rotation, avoiding the volume distortion or asymmetric bulging that may occur with laterally based flaps. As a result, AICAP can recreate a near-native breast shape and feel using a minimal incision—an advantage that is particularly apparent in patients with small-to-moderate breast volume. Compared with LICAP and LTAP, AICAP places the incision more anteriorly and inferiorly, allowing it to be hidden within the IMF in most cases rather than lying along the anterior or posterior axillary line. This distinction—scar present but unseen—is especially meaningful in East Asian women, whose body habitus and aesthetic expectations prioritize subtlety in scar location. Moreover, the donor-tissue properties closely resemble those of the breast, enabling postoperative consistency in vascularity and skin tone. This avoids the noticeable color mismatch and texture discordance often seen with traditional local flaps (12, 55).

Improvements in breast contour translate directly into enhanced quality of life. Long-term follow-up data from Dahlbäck et al. (59) using BREAST-Q and BCCT.core demonstrated that aesthetic scores correlate positively with psychological well-being, social confidence, and overall quality of life, indicating that favorable breast appearance provides sustained and meaningful benefits. Findings specific to AICAP support this trend. Carrasco-López et al. (12) observed significant postoperative gains in the BREAST-Q domains of “breast satisfaction” and “physical well-being,” with patients achieving psychological benefits not present preoperatively. These advantages have been validated in larger populations. Multicenter studies of CWPF techniques have shown that more than 80% of patients receive “good to excellent” aesthetic ratings postoperatively (44). In another series evaluating local perforator flaps—including AICAP—Zeeshan et al. (17) reported a Rasch-converted BREAST-Q score of 100 for “breast satisfaction” and 76 for “physical well-being,” with very low complication rates, underscoring that local perforator flaps not only “repair a defect” but provide high-quality aesthetic outcomes with minimal risk. Autologous reconstruction also offers clearer long-term advantages over implant-based reconstruction. Implants are frequently associated with capsular contracture, firmness, and foreign-body sensation, whereas AICAP reconstruction—using the patient’s own tissue—maintains a natural feel and pliability over time. Findings from the MROC multicenter study (60) further confirmed that autologous reconstruction surpasses implant-based approaches in long-term “breast satisfaction,” “psychosocial well-being,” and “physical well-being.” Taken together, these findings indicate that AICAP, as an autologous reconstruction option within the CWPF family, is not only safe and feasible but also capable of delivering sustained aesthetic and quality-of-life benefits over long-term follow-up.

It is particularly noteworthy that AICAP demonstrates unique contour stability in the setting of adjuvant radiotherapy. Radiation therapy often induces fibrosis, microvascular damage, and progressive changes in breast shape; however, because AICAP provides an independent vascular supply and does not involve an implant capsule, its long-term volume and contour maintenance are markedly superior to implants or certain non-anatomic local flaps. In a cohort of 100 patients undergoing local perforator flap reconstruction, Roy et al. (61) reported stable breast symmetry at a median follow-up of eight years, despite a high rate of postoperative irradiation. Similarly, a systematic review by Nava et al. (26) confirmed that among irradiated patients, muscle-preserving flaps based on local perforators did not exhibit increased risk of volume loss or contour deformity. Given that the lower-inner quadrant is particularly vulnerable to radiation-induced deformity, AICAP’s anatomical location inherently bypasses this risk. This positioning makes AICAP not merely an alternative to lateral flaps but a potentially preferred option for reconstruction in “trouble quadrants,” where aesthetic and structural challenges are greatest.

In summary, AICAP achieves high aesthetic scores and strong patient satisfaction through a combination of precise anatomical conformity, well-concealed scarring, natural volume replacement, and excellent tolerance to radiotherapy. It offers a refined autologous reconstruction pathway with distinct advantages for breast-conserving surgery. Across both oncologic safety and aesthetic performance, AICAP stands alongside LICAP and LTAP as an established technique, while providing a clear additional benefit for defects in the lower-inner quadrant.

## 7 Limitations and challenges

Despite the clear anatomical advantages and favorable aesthetic potential of the AICAP flap in reconstruction of inferomedial breast defects, its clinical application remains constrained by several factors. Foremost among these is the limited level of evidence and relatively short duration of follow-up. The existing literature on AICAP largely consists of single-center, small-sample retrospective or prospective case series, with median follow-up periods typically limited to 2–3 years (12, 55, 62, 63). Moreover, outcome reporting lacks standardization, particularly with respect to long-term oncologic endpoints—such as disease-free survival (DFS) and overall survival (OS)—and patient-reported outcome measures (PROMs) beyond five years. Consequently, although current data suggest favorable short-term aesthetic outcomes and acceptable perioperative safety, the long-term oncologic safety and sustained patient benefit of AICAP reconstruction require confirmation through longer follow-up and more standardized outcome frameworks (26).

In addition, the indications and available donor-tissue volume of AICAP are inherently limited. This flap is primarily suited for small- to moderate-volume defects of the inferomedial or subareolar region, generally not exceeding approximately 30% of total breast volume. When defects are larger or located in the upper outer quadrant, AICAP alone often fails to provide sufficient soft-tissue volume and lower-pole support, potentially resulting in inferior pole flattening or suboptimal contour. In such scenarios, combined approaches incorporating LICAP or LTAP flaps, or multimodal strategies involving implants and/or fat grafting, are more commonly favored (12, 55). Furthermore, substantial interindividual variability in the number, caliber, and emergence points of anterior intercostal perforators increases technical complexity and hinders procedural standardization. Even with preoperative localization using handheld Doppler or ultrasonography, some perforators may prove unsuitable intraoperatively due to insufficient caliber or compromised perfusion (21, 44), thereby increasing reliance on surgeon experience and intraoperative judgment (23, 62).

From another perspective, AICAP reconstruction is an open, invasive technique, and donor-site scar management and associated complications warrant careful consideration. Although incisions are typically placed along the inframammary fold and concealed by undergarments, scar length, color mismatch, and subtle contour changes may still affect subjective satisfaction, particularly in slender patients or those with heightened aesthetic sensitivity. Excessive flap harvest or inadequate tension control may further increase the risk of fat necrosis, marginal ischemia, or delayed wound healing (12), with heightened vulnerability observed in smokers, obese patients, and individuals with diabetes (64). While robotic or endoscopic-assisted techniques have been proposed as potential means to reduce donor-site morbidity and optimize scar concealment, their application in AICAP reconstruction remains limited to small series or technical reports (65). As such, the true long-term clinical value of these emerging approaches has yet to be established through robust, evidence-based investigation.

## 8 Future perspectives

Current evidence indicates that AICAP flaps can achieve negative margins, low complication rates, and favorable aesthetic outcomes in immediate reconstruction of lower-pole and inferomedial defects following breast-conserving surgery, without delaying adjuvant radiotherapy. Nevertheless, several critical issues must be addressed systematically before wider adoption can be justified.

First, standardization of preoperative imaging assessment remains fundamental to improving the reproducibility of AICAP reconstruction. At present, perforator localization relies predominantly on handheld Doppler, despite prior studies demonstrating its limited and inconsistent detection of anterior intercostal perforators. In contrast, CTA provides more accurate visualization of perforator distribution and identification of potential dominant zones, thereby reducing the risk of intraoperative flap redesign and marginal perfusion compromise (12). Building on this, future work should explore integration of CTA with automated perforator recognition algorithms to establish a more structured preoperative planning pathway, reducing dependence on individual surgeon experience and improving operative efficiency (66). In addition, CTA-based three-dimensional reconstruction and patient-specific preoperative planning have shown promise in shortening operative time and enhancing procedural consistency in other perforator flaps, and their applicability to AICAP reconstruction warrants further evaluation (67).

Second, the clinical role of AICAP should be embedded within a broader chest wall perforator decision framework. In recent years, several studies have explored the use of AICAP in combination with implant coverage, lower-pole reconstruction, or repair of larger excisional defects, suggesting that this flap should not be regarded solely as a localized volume replacement technique but rather as a complementary option alongside LICAP, LTAP, and SEAP flaps. In specific scenarios—namely inferomedial defects requiring concealed scarring and postoperative radiotherapy—clear prioritization of AICAP indications may facilitate more refined, individualized reconstructive strategies (22). Meanwhile, reports of mild inframammary fold distortion or the need for medial contour adjustment following AICAP reconstruction underscore the importance of continued technical refinement and combined approaches to enhance long-term shape stability (68).

Most importantly, follow-up durations in the current AICAP literature remain limited, with median follow-up in most series falling short of 36 months. Recent multicenter studies on chest wall perforator flaps have adopted disease-free survival, overall survival, and patient-reported outcome measures (PROMs) as primary endpoints (33), highlighting the need for future AICAP-focused research to progress toward prospective, multicenter designs with follow-up of at least five years. Incorporation of standardized oncologic and aesthetic outcome measures will be essential to enable meaningful comparisons with other chest wall perforator techniques.

Finally, as endoscopic and robotic-assisted approaches gain traction in breast reconstruction, their potential role in anterior intercostal perforator exposure merits investigation, particularly with regard to minimizing donor-site incisions and further optimizing scar concealment. Overall, future AICAP research should transition from feasibility and safety validation toward standardized clinical implementation, encompassing structured preoperative assessment, quantitative indication criteria, and systematic evaluation of long-term outcomes. With accumulation of high-quality evidence and refinement of clinical workflows, AICAP has the potential to evolve into a reproducible and widely applicable option in oncoplastic breast reconstruction.

## 9 Conclusion

AICAP represents an emerging direction in oncoplastic breast surgery, offering aesthetic reconstruction while maintaining oncologic safety. Its anatomical location adjacent to the lower and lower-inner quadrants, combined with reliable perfusion and preservation of pectoralis major function, enables precise volume replacement without compromising donor-site function. Current evidence demonstrates that AICAP reconstruction achieves high negative-margin rates, low complication rates, favorable aesthetic outcomes, and does not delay adjuvant radiotherapy. Compared with traditional myocutaneous flaps or implant-based reconstruction, AICAP provides minimal morbidity, a more natural breast contour, and superior post-radiation stability—features particularly advantageous for Asian women with small-to-moderate breast volume. However, clinical standardization of the technique remains in its early stages. Anatomical variability of the perforators, limited long-term oncologic follow-up, and the absence of unified imaging protocols for preoperative localization all restrict broader adoption. Future work should prioritize the development of comprehensive anatomical databases and CTA-based perforator mapping algorithms to enhance preoperative predictability. Integrating AI-assisted CTA analysis with patient-specific 3D-printed models may further optimize surgical planning and shorten the learning curve. Additionally, incorporating robotic and endoscopic-assisted techniques holds promise for reducing donor-site incision size, improving scar concealment, and refining aesthetic precision. In summary, the AICAP flap offers a reproducible, aesthetically favorable option for partial breast reconstruction following breast-conserving surgery. With continued advancements in imaging, digital surgical planning, and minimally invasive technologies, AICAP is well positioned to evolve from an experience-dependent approach into a standardized, widely applicable oncoplastic reconstructive strategy—achieving a true balance between oncologic radicality and aesthetic restoration.
